# The use of Silodosin in Ureterolithiasis is the Hot Topic in this Number of International Brazilian Journal of Urology

**DOI:** 10.1590/S1677-5538.IBJU.2026.02.01

**Published:** 2026-03-16

**Authors:** Luciano A. Favorito

**Affiliations:** 1 Universidade do Estado do Rio de Janeiro Unidade de Pesquisa Urogenital Rio de Janeiro RJ Brasil Unidade de Pesquisa Urogenital - Universidade do Estado do Rio de Janeiro - Uerj, Rio de Janeiro, RJ, Brasil; 2 Hospital Federal da Lagoa Serviço de Urologia Rio de Janeiro RJ Brasil Serviço de Urologia, Hospital Federal da Lagoa, Rio de Janeiro, RJ, Brasil

The March-April number of International Brazilian Journal of Urology presents original contributions with a lot of interesting papers in different fields: Robotic Surgery, Artificial Intelligence, BPH, Endourology, Kidney cancer, Basic Research, Infertility, Bladder Cancer, Micropenis, Prostate Cancer and Reconstructive Urology. The papers came from many different countries such as Brazil, Italy, China, Denmark, Colombia, United Kingdom and Germany, and as usual the editor's comment highlights some of them. The editor in chief would like to highlight the works about Silodosin.

Dr. Godinho and collegues from Brazil, presented in page e20250355 (1) a nice systematic review about the "Impact of preoperative Silodosin on ureteroscopy outcomes for ureterolithiasis". The paper shows important aspects about the α-adrenergic receptor antagonists and their capacity to optimize preoperative conditions during ureteroscopy. Ureteroscopy (URS) is one of the most important modalities for the management of ureteral stones (2-6). Silodosin is a selective alpha-1A adrenergic receptor blocker has at least one registered product approved for commercialization in Brazil by ANVISA in 2025.

In the present paper the authors concluded that the use of silodosin as a preoperative treatment in the URS approach for ureterolithiasis improves both the safety and efficiency of the procedure compared to no preoperative therapy. Future research should prioritize randomized controlled trials that incorporate stratification based on stone location while also focusing on standardizing the definition of tone-free rate, ensuring proper follow-up, and optimizing preoperative Silodosin treatment duration

The Editor-in-chief expects everyone to enjoy reading.
